# Voltage-gated sodium channels and cancer: is excitability their primary role?

**DOI:** 10.3389/fphar.2015.00152

**Published:** 2015-07-29

**Authors:** Sébastien Roger, Ludovic Gillet, Jean-Yves Le Guennec, Pierre Besson

**Affiliations:** ^1^Inserm UMR1069, Nutrition, Croissance et Cancer, Université François-Rabelais de ToursTours, France; ^2^Département de Physiologie Animale, UFR Sciences and Techniques, Université François-Rabelais de ToursTours, France; ^3^Department of Clinical Research, University of BernBern, Switzerland; ^4^Inserm U1046, Université de MontpellierMontpellier, France

**Keywords:** voltage-gated sodium channel, cell excitability, cancer, sodium, invasion

## Abstract

Voltage-gated sodium channels (Na_V_) are molecular characteristics of excitable cells. Their activation, triggered by membrane depolarization, generates transient sodium currents that initiate action potentials in neurons and muscle cells. Sodium currents were discovered by Hodgkin and Huxley using the voltage clamp technique and reported in their landmark series of papers in 1952. It was only in the 1980's that sodium channel proteins from excitable membranes were molecularly characterized by Catterall and his collaborators. Non-excitable cells can also express Na_V_ channels in physiological conditions as well as in pathological conditions. These Na_V_ channels can sustain biological roles that are not related to the generation of action potentials. Interestingly, it is likely that the abnormal expression of Na_V_ in pathological tissues can reflect the re-expression of a fetal phenotype. This is especially true in epithelial cancer cells for which these channels have been identified and sodium currents recorded, while it was not the case for cells from the cognate normal tissues. In cancers, the functional activity of Na_V_ appeared to be involved in regulating the proliferative, migrative, and invasive properties of cells. This review is aimed at addressing the non-excitable roles of Na_V_ channels with a specific emphasis in the regulation of cancer cell biology.

## When exciting discoveries hide non-excitable functions

In the mind of every biologist, the voltage-dependent activation of sodium currents, carrying inward charges, and responsible for membrane depolarization, is critical and is a specificity of cells characterized as being “excitable,” such as neurons, skeletal, and cardiac muscle cells. This knowledge on electrophysiology and cellular excitability directly comes from the tremendous work of Hodgkin and Huxley, co-laureates of the Nobel Prize in Physiology and Medicine with Eccles in 1963, for their study of the action potential and the ionic mechanisms involved in nerve cell membrane excitation. Indeed, in a series of studies they published in 1952, they reported the analysis of action potential in the squid giant axon and showed that electrical signals are initiated by sodium currents being activated after an initial membrane depolarization (Hodgkin and Huxley, 1952a,b,c,d,e; Hodgkin et al., 1952).

Sodium currents (I_Na_) generate the rapid depolarization phase of the action potential. They are transient and their fast inactivation occurring within 1–2 ms, along with the delayed activation of voltage-gated potassium currents, allows the membrane repolarization and the termination of the electrical signal. While this “sodium activity” was identified and recorded using voltage-clamp techniques, nothing was known at that time on the structure and regulation of membrane proteins responsible for this sodium permeation. The identification, cloning and purification of voltage-gated sodium channel proteins (Na_V_) came almost 30 years later, in the 1980's thanks to the works performed in the respective laboratories of Catterall and of Numa (for review, see Catterall, 2012). These studies, along with the multiple mutagenesis studies that were performed, permitted to propose structural models supporting the different properties of Na_V_ channels (ion selectivity and conductance, activation, and inactivation) that were more recently clarified by information obtained from the X-ray diffraction of crystal structures of two bacterial Na_V_ channels from *Arcobacter butzleri* (NavAb) and from *Magnetococcus* sp. *marinus* strain MC-1 (NavMs). NavAb structure was studied in a hybrid closed-pore conformation but with four activated voltage sensors (Payandeh et al., 2011), then in two potential inactivated states (that are more related to the slow inactivation found in vertebrate channels since bacterial channels do not have the fast inactivation) (Payandeh et al., 2012), while NavMs was studied in an open conformation (McCusker et al., 2012).

It is now well-established that, in mammals, voltage-gated sodium channels are multimeric transmembrane complexes composed of a large pore-forming α subunit (Na_V_α) associated with one or two, identical or different, smaller β subunits (Na_V_β) (Catterall, 2000; Brackenbury and Isom, 2011).

There are nine genes in humans (*SCN1A, SCN2A, SCN3A, SCN4A, SCN5A, SCN8A, SCN9A, SCN10A*, and *SCN11A*) which encode for nine different Na_V_α proteins (Na_V_1.1 to Na_V_1.9, respectively). Na_V_α are sensitive to membrane depolarization and belong to a single family owing to their high level of homology (Goldin et al., 2000). These Na_V_α proteins are all composed of four homologous domains (I–IV), each containing six α-helical transmembrane domains (S1–S6) (Noda et al., 1984). The first four segments (S1–S4) of each domain are proposed to constitute the voltage-sensing domain (VSD), and the two other segments separated by the reentrant extracellular P loop (S5-P-S6) form the pore of the channel (Payandeh et al., 2011). Sodium currents can be inhibited by many drugs and toxins that interact with Na_V_α proteins at different binding sites and the well-known selective inhibitor is the pore-blocking toxin tetrodotoxin (TTX) that interacts with amino acid residues controlling Na^+^ selectivity in the short α-helical segment of the P-loop between S5 and S6 (Noda et al., 1989; Terlau et al., 1991; Heinemann et al., 1992). While all Na_V_α can be inhibited by TTX, two families of channels have been identified as a function of their sensitivity to TTX: the “TTX-sensitive channels” Na_V_1.1-1.4, Na_V_1.6, and Na_V_1.7 that can be blocked by nanomolar concentrations, and the so-called “TTX-resistant channels” Na_V_1.5, Na_V_1.8, and Na_V_1.9 requiring micromolar concentrations to be blocked. Na_V_1.8 channel is the most insensitive as it has a TTX IC_50_≥50 μM (Catterall et al., 2003).

Obviously, the expression and activity of such proteins were particularly studied in excitable cells and a preferential tissue distribution was described as following: Na_V_1.1–1.3 and Na_V_1.6 being central nervous system (CNS) neuronal channels, Na_V_1.7–1.9 being the primary channels of sensory, sympathetic, and nociceptive neurons of the peripheral nervous system (PNS), whereas the Na_V_1.4 channel is recognized to be the primary skeletal muscle channel and Na_V_1.5 being the predominant channel of the cardiac muscle. However, some tissues express multiple isoforms of the channels at various levels and in specific subcellular domains at the plasma membrane. For example, Na_V_1.5 is the predominant channel expressed in cardiac cells, proposed to be responsible for the transient sodium current (also called I_NaT_) responsible for the initial membrane depolarization during the cardiac action potential. However, other channels, initially called “non-cardiac channels” have been found to be expressed in the heart, albeit to a lesser extent than Na_V_1.5. These comprise Na_V_1.1, Na_V_1.2, Na_V_1.3, Na_V_1.4, Na_V_1.6, Na_V_1.7, and Na_V_1.8 channels (Maier et al., 2004; Haufe et al., 2005a,b; Marionneau et al., 2005; Gershome et al., 2011; Noujaim et al., 2012; Yang et al., 2012b). These “non-cardiac” channels have been proposed to be mainly expressed at T-tubules and at the lateral membranes of cardiomyocytes (Brette and Orchard, 2006; Lin et al., 2011) or even in intracardiac neurons participating to the neural regulation of the cardiac function (Maier et al., 2002; Verkerk et al., 2012; Westenbroek et al., 2013). While the role and importance of these non-cardiac channels in the physiological cardiomyocyte function is not fully characterized, they may be responsible for a persistent (or late) sodium current (called I_NaP_) (Biet et al., 2012) and involved in some pathological conditions leading to arrhythmias (Yang et al., 2012b; Mishra et al., 2015).

Mutations occurring in genes encoding Na_V_α may lead to inherited pathologies called “sodium channelopathies.” These mutations are responsible for genetically dominant neurological, muscular or cardiac disorders, and have been characterized by abnormal excitability. The majority of these mutations cause ***gain-of-function*** effects by impairing Na_V_ channel (fast or slow) inactivation and prolonging the entry of Na^+^ ions into the cells. This is the case for gain-of-function mutations of Na_V_1.5 resulting in a prolonged ventricular action potential that have been associated with LQT3, a syndrome characterized by a prolonged Q-T interval on the electrocardiogram, and responsible for cardiac arrhythmias (Wang et al., 1995a,b; Keating and Sanguinetti, 2001). Mutations in Na_V_1.4 resulting in inactivation impairments have been associated with skeletal myopathies in apparently opposing effects such as hyperkalaemic periodic paralysis characterized by muscular hypoexcitability, or even paramyotonia congenita or potassium-aggravated myotonia for which patients suffer from periods of muscular hyperexcitability, with retarded relaxation and spontaneous firing of action potentials, which can be followed by hypoexcitability periods (Jurkat-Rott et al., 2010). These striking differences depend in fact on the proportion of non-inactivating channels: while a low proportion of non-inactivated channels can lead to muscular hyperexcitability, a high proportion of non-inactivated channels rapidly generates paralysis (Hayward et al., 1996). Gain-of-function mutations have been identified in Na_V_1.7 channels expressed in small-diameter dorsal root sensory neurons and cause severe painful neuropathies, such as in erythromelalgia, due to the hyperpolarization shift of the voltage dependence of activation or an impaired inactivation (Waxman et al., 2014; Hoeijmakers et al., 2015). *Loss-of-function* mutations have also been identified in these channels, such as in Na_V_1.5 in Brugada syndrome, thus generating arrhythmias due to inhomogeneous electrical conduction in ventricles (Remme, 2013) or in Na_V_1.7 causing rare recessive congenital loss of pain sensation (Cox et al., 2006).

There are five Na_V_β subunits, β1, β1B, β2, β3, and β4, which are encoded by four different genes. Subunits β1 and β1B are splice variants encoded by the same *SCN1B* gene (Isom et al., 1992; Kazen-Gillespie et al., 2000; Qin et al., 2003), while β2 (Isom et al., 1995), β3 (Morgan et al., 2000), and β4 (Yu et al., 2003) are encoded by *SCN2B, SCN3B*, and *SCN4B* genes, respectively. All five Na_V_β have an extracellular N-terminal region containing an Immunoglobulin (Ig) domain, homologous to V-type Ig loop motifs, which is maintained by two conserved cysteine residues. With the exception of β1B, all Na_V_β subunits are transmembrane proteins that have a single α-helical transmembrane domain and a short intracellular domain (Brackenbury and Isom, 2011). β1B, initially called β1A, is due to an alternative splicing retaining intron three in *SCN1B* gene. This results in a protein that differs from β1 by the absence of a C-terminal transmembrane domain (Qin et al., 2003). Therefore, β1B is the only member of the Na_V_β family to be a soluble and secreted protein (Kazen-Gillespie et al., 2000; Patino et al., 2011). Na_V_β subunits are non-pore forming proteins that were initially isolated from rat brain along with Na_V_α (Messner and Catterall, 1985). From this pioneer work, they have been proposed to be auxiliary of Na_V_α, and they were indeed demonstrated to promote Na_V_α trafficking to the plasma membrane as well as modulation of the voltage-dependence of activation and inactivation, the rate of inactivation, the recovery from inactivation and the presence of persistent or resurgent currents (Calhoun and Isom, 2014). They were also reported to modulate the pharmacology of Na_V_α, such as the sensitivity to lidocaine (Lenkowski et al., 2003) or the binding affinity of some conotoxins (Wilson et al., 2011; Zhang et al., 2013). Na_V_α and Na_V_β subunits can interact physically. Indeed, β1 and β3 proteins interact with Na_V_α through non-covalent associations within both their N- and C-termini (McCormick et al., 1998; Meadows et al., 2001), while β2 and β4 proteins covalently interact with Na_V_α through the formation of a disulfide bond, thanks to a conserved cysteine residue present in the N-terminal extracellular Ig loop (Chen et al., 2012; Gilchrist et al., 2013). Recently, the structures of the extracellular domains of human β3 and β4 have been solved at the atomic level by X-ray crystallography bringing new insights on the interactions and stoichiometry between Na_V_α and Na_V_β, but also between Na_V_β subunits themselves, as β3 was proposed to form trimers (Gilchrist et al., 2013; Namadurai et al., 2014). Besides this well-known role in regulating Na_V_α addressing to the plasma membrane and activity, Na_V_β subunits were proposed to achieve other cellular functions. Indeed, the presence of an Ig motif in their extracellular domain suggested a possible role of these proteins as *cis* and *trans* cell adhesion molecules (CAMs), similar to that of integrins, cadherins, and selectins (Isom and Catterall, 1996; Isom, 2002). β1 and β2 subunits have been demonstrated to form both *trans*-homophilic and *trans*-heterophilic cell-cell and cell-matrix adhesions, while it is more controversial for β3, and unclear for β4. These adhesions have been mainly studied in cells expressing Na_V_α, such as neurons in which they were proven to be critical for neurites outgrowth, axonal fasciculation and interactions with glial cells (O'malley and Isom, 2015). Several mutations have also been identified in genes encoding Na_V_β, and have been associated with a number of diseases primarily occurring in excitable tissues, such as epilepsies, cardiac arrhythmias and sudden death, due to the disturbed modulation of Na_V_α and cell excitability (see a recent review O'malley and Isom, 2015). For example, a missense mutation in the *SCN4B* gene, resulting in the L1759F amino acid substitution in the β4 protein, has been associated with LQT10. This mutation induces a gain-of-function of Na_V_1.5, with a depolarizing shift of the voltage-dependence of inactivation, therefore leading to an increased window current and an increased I_NaP_ (Medeiros-Domingo et al., 2007).

Thus mutations or altered expression of Na_V_β could lead to pathologies due to the regulation of Na_V_α functioning. Alternatively, the possibility cannot be excluded that pathologies could arise from Na_V_β cell adhesion properties *per se*.

Besides these relatively well-characterized functions of Na_V_ channels in excitable cells, multiple studies have demonstrated the functional expression of Na_V_α in normal non-excitable cells, such as in macroglial cells [astrocytes (Sontheimer and Waxman, 1992), oligodendrocytes (Tong et al., 2009), Schwann cells (Chiu et al., 1984)], in immune cells [microglia (Korotzer and Cotman, 1992; Craner et al., 2005), dendritic cells (Kis-Toth et al., 2011), macrophages (Carrithers et al., 2007, 2009), T-lymphocytes (Decoursey et al., 1985; Fraser et al., 2004)], endothelial cells (Traub et al., 1999; Andrikopoulos et al., 2011), osteoblasts (Black et al., 1995), odontoblasts (Allard et al., 2006), chondrocytes (Sugimoto et al., 1996), keratinocytes (Zhao et al., 2008), and fibroblasts (Chatelier et al., 2012). While their physiological roles were not clearly demonstrated so far, they were proposed to regulate cellular functions such as survival or proliferation, cell migration, cell differentiation, endosome acidification, phagocytosis, and podosome formation. This was recently reviewed by Black and Waxman and referred to as the “non-canonical roles” of Na_V_ (Black and Waxman, 2013). As such, it becomes clearer that Na_V_α may have developmental and physiological roles that have been underestimated for long and while being associated to channel activities and sodium permeation, are not linked to the generation of action potentials. This naturally raises the questions on the mechanisms by which Na_V_ channels fulfill their physiological roles, and how the voltage-dependence could represent a selective advantage in non-excitable cells.

Another aspect of interest is the “abnormal” expression of Na_V_ channels in non-excitable cancer cells, and especially in carcinoma cells. This new interest originates from studies in human and rodent cell lines and biopsies, in which both Na_V_α and Na_V_β have been reported and sodium currents were recorded. The abnormal expression of Na_V_α and Na_V_β proteins and particularly the activity of Na_V_α in carcinoma cells were very often, if not always, associated with aggressive features, and it is tempting to consider cancer as a (sodium) channelopathy. In this article, we wish to review the present knowledge on the expression of Na_V_α and Na_V_β in cancer cells and discuss the possible mechanisms by which this gain-of-function could regulate oncogenic properties in comparison with their roles in non-excitable cells.

## Expression of voltage-gated sodium channels in cancer cells: The potential for an action in oncogenic properties

Cancers, which are most of the time sporadic genetic diseases, are among the leading causes of death in the world. The most common cancers are carcinomas, originating from epithelial tissues, such as lung, breast, prostate, cervix or colon cancers, and are responsible for the high mortality by cancer (Parkin et al., 2005). While cancers differs in their incidence and mortality rate, depending on the organs in which the primary tumor appears, the cancer disease is always the result of mutual interactions between cancer cells from the tumor and the host organism. As such, the disease and its progression are consequences of very complex interplays between cancer cells and non-cancer cells (Hanahan and Weinberg, 2000). Obviously, the promoter of such a situation is the cancer cell itself, having accumulated multiple genomic mutations and having survived, as in the Darwinian principle of evolution, despite the high pressure of selection in the environment. Indeed, selected cancer cells have gained proliferative advantages since they had to resist to cell death, to escape the immune system control, to thrive under the paucity of stimulating growth factors or despite the existence of dampening signaling pathways, to survive to episodes of O_2_ and nutrients deprivation, etc. According to this stringent process, the vast majority of cancer cells are dying, but those that are surviving to environmental constraints have been selected on the basis of transcriptional modulations that conferred immortality, growth and invasion advantages. This leads to the well-known phenotype of cancer cells that have been defined, by Hanahan and Weinberg, as the acquisition of eight biological capacities: self-sufficiency for proliferative signaling, insensitivity to anti-growth signals, resistance to apoptosis, replicative immortality, energy metabolism adapted to hypoxia, stealth to immune detection and destruction, induction of tumor angiogenesis, activated extracellular matrix invasion, and metastasis (Hanahan and Weinberg, 2011). Therefore, the selected cancer cells are the most fitted to the drastic environment and definitely the most aggressive, because of the repression of some growth-limiting genes and the overexpression of some other genes that are definitely associated with aggressiveness. Except in the small proportion of cases for which there are mutations in initiation genes (such as in *BRCA1* for example), the genes that are deregulated in cancer cells are not different from those found in normal cells. The signaling pathways that are regulated by the deregulated genes should be seen as misappropriations by cancer cells of physiologically regulated functions. These functions could have been already described or may still be unknown. In this idea, the carcinogenetic process is very often compared with conditions such as embryonic development and tissue repairing, with the ultimate difference that these latter processes are fully controlled and are always ended-up by cell differentiation.

Over the last two decades, numerous studies have reported the abnormal overexpression, or underexpression, of some ion channels in cancer cells, as compared with corresponding non-cancer cells. Because plasma membrane ion channels are transmembrane proteins that generate ion fluxes, they are key regulators of ion homeostasis, membrane potential, cell volume, and intracellular signaling events. Their abnormal function was found to regulate several cancer cell biological processes, such as cell proliferation, resistance to apoptosis, cell adhesion, cancer cell motility, or extracellular matrix invasion (Schwab and Stock, 2014; Litan and Langhans, 2015). As such some ion channels were proposed to represent the “hallmarks of cancer cells” (Prevarskaya et al., 2010). This is certainly the case for Na_V_ proteins which have been reported to be highly overexpressed in cancer biopsies and cancer cells while they are undetectable in most cognate normal tissues (see Table 1) and proposed to serve as new targets for cancer therapy (Roger et al., 2006).

**Table 1:** **List of the non-nervous tissue cancers in which Na_V_α have been studied**.

**Tissues**	**Cancer/Non-cancer**	**Cell lines or biopsies**	**mRNA Expression**	**Protein in cell line or biopsies**	**TTX sensitivity**	**Functional expression**	**Na_V_α Role**	**Na_V_α in biopsies**	**References**
Prostate	Cancer	Biopsies		Na_V_1.8				Yes (IH); staining for Na_V_1.8 correlated with Gleason score: very low in normal epithelium, low in moderately aggressive stages, strong in highly aggressive cancer with appearance of staining in nucleus	Suy et al., 2012
		PC-3	Na_V_1.2 Na_V_1.3 Na_V_1.7	Na_V_1.1 Na_V_1.2 Na_V_1.5 Na_V_1.6 Na_V_1.7 Na_V_1.8 Na_V_1.9	8.6 nM	nNa_V_1.7	Invasion Motility (galvanotaxis) Motility (wound healing)		Laniado et al., 1997; Diss et al., 2001; Abdul and Hoosein, 2002; Suy et al., 2012
		22Rv1		Na_V_1.1 Na_V_1.2 Na_V_1.5 Na_V_1.6 Na_V_1.8 Na_V_1.9					Suy et al., 2012
		DU-145		Na_V_1.1 Na_V_1.2 Na_V_1.5 Na_V_1.6 Na_V_1.7 Na_V_1.8 Na_V_1.9					Suy et al., 2012
		LnCaP	Na_V_1.2	Na_V_1.1 Na_V_1.2 Na_V_1.5 Na_V_1.6 Na_V_1.7 Na_V_1.8 Na_V_1.9		No current			Laniado et al., 1997; Diss et al., 2001; Suy et al., 2012
		Mat-Ly-Lu (rat)	Na_V_1.1 Na_V_1.4 Na_V_1.7		18 nM	nNa_V_1.7	Invasion (galvanotaxis) Motility (wound healing)		Grimes et al., 1995; Grimes and Djamgoz, 1998; Diss et al., 2001
		AT-2 (rat)	Na_V_1.1 Na_V_1.4 Na_V_1.9			No current	—	—	Grimes et al., 1995; Diss et al., 2001
	Non-Cancer	PNT2 (normal, immortalized)	?			No current	—	—	Mycielska and Djamgoz, 2004
Breast	Cancer	Biopsies	Na_V_1.5 Na_V_1.6 Na_V_1.7					Yes (IH, RT-PCR)	Fraser et al., 2005
		MDA-MB-231	fNa_V_1.5 Na_V_1.6 Na_V_1.7		~2 μM	nNa_V_1.5	*In vitro* invasion Extracellular acidification through allosteric enhancement of NHE1 activity;	Yes (RT-PCR)	Roger et al., 2003; Fraser et al., 2005; Brisson et al., 2011, 2013;
							promotion of proteolytic invadopodial activity Promotion of metastasis in immunodeficient mice		Driffort et al., 2014; Nelson et al., 2015
		MCF-7	Na_V_1.5 Na_V_1.6 Na_V_1.7			No current	—	—	Roger et al., 2003
		MDA-MB-468	?			No current			Roger et al., 2003
Lung	Small-cell lung cancer	H146	?		215 nM	Current	?	?	Pancrazio et al., 1989; Blandino et al., 1995
		H128	?		?	Current	?	?	Pancrazio et al., 1989
		H69	Na_V_1.3 Na_V_1.5 Na_V_1.6		TTX-S	Current	Endocytosis	?	Pancrazio et al., 1989; Onganer and Djamgoz, 2005
		H209	Na_V_1.3 Na_V_1.5 Na_V_1.6		TTX-S		Endocytosis	?	Onganer and Djamgoz, 2005
		H510	Na_V_1.3 Na_V_1.5 Na_V_1.6 Na_V_1.9		TTX-S		Endocytosis	?	Onganer and Djamgoz, 2005
	Non-small cell lung cancer	H460	Na_V_1.3 Na_V_1.5 Na_V_1.6 Na_V_1.7		~10 nM	Na_V_1.7	Invasion		Roger et al., 2007; Campbell et al., 2013
		Calu-1	Na_V_1.1 Na_V_1.2 Na_V_1.3 Na_V_1.5 Na_V_1.6 Na_V_1.7 Na_V_1.8 Na_V_1.9		~5 nM + ~1 μM	Na_V_1.1 Na_V_1.2 Na_V_1.3 Na_V_1.5 Na_V_1.6 Na_V_1.7	Invasion		Roger et al., 2007
		H23	Na_V_1.5 Na_V_1.6 Na_V_1.7		~10 nM	Na_V_1.6 Na_V_1.7	Invasion		Roger et al., 2007
		A549	Na_V_1.6 Na_V_1.7			No current	—	—	Roger et al., 2007
	Non-cancer	NL20 (normal, immortalized)	Na_V_1.2 Na_V_1.3 Na_V_1.6 Na_V_1.7			No current	—	—	Roger et al., 2007
		BEAS-2B (normal, immortalized)	Na_V_1.1 Na_V_1.5 Na_V_1.6 Na_V_1.7			No current	—	—	Roger et al., 2007
Leukocytes	Leukemia	K562	?		?	Current (type not identified)	?	?	Schlichter et al., 1986
		K562				No current	—	—	Yamashita et al., 1987
		K562/ADM	?		< 100 nM	Current (type not identified)	?	?	Yamashita et al., 1987
		CCRF-CEM	?		< 150 nM	Current (type not identified)	?	?	Lee et al., 1988
		CEM/VLB100	?		< 150 nM	Current (type not identified)	?	?	Lee et al., 1988
		Jurkat (normal, immortalized)	Na_V_1.5 Na_V_1.6 Na_V_1.7 Na_V_1.8 Na_V_1.9		~900 nM	Na_V_1.5 Na_V_1.7	invasion		Fraser et al., 2004
	Normal	Normal human lymphocytes				Current (type not identified) in three cells out of 90			Cahalan et al., 1985
		Primary-cultured human monocytes-derived macrophages	Na_V_1.5 Na_V_1.6	Na_V_1.5 (IH)Na_V_1.6 (IH)	TTX-R	Na_V_1.5; protein localized in late endosomes	Phagocytosis, acidification of endosomes		Carrithers et al., 2007
Pleura	Mesothelioma	Primary-cultured human malignant pleural mesothelioma cells	Na_V_1.2 Na_V_1.3 Na_V_1.4 Na_V_1.5 Na_V_1.6 Na_V_1.7			TTX-sensitive current	Increases *in vitro* motility		Fulgenzi et al., 2006
	Normal mesothelial cells	(Normal, immortalized)				No current			Fulgenzi et al., 2006
Cervix	Cancer	Primary-cultured human cervical carcinoma cells	Na_V_1.1 Na_V_1.2 Na_V_1.3 Na_V_1.4 Na_V_1.6 Na_V_1.7a Na_V_1.7b	Na_V_1.6 (IH)Na_V_1.7 (IH)		TTX-sensitive current	Na_V_1.6-dependent invasion		Diaz et al., 2007
	Non-cancer	(Biopsies of normal uterine cervix)	Na_V_1.1 (very low) Na_V_1.2 Na_V_1.3 Na_V_1.4 Na_V_1.6 (very low)Na_V_1.7a Na_V_1.7b (very low)	Na_V_1.6 (IH)Na_V_1.7 (IH)					Diaz et al., 2007
Colon	Cancer	Biopsies		Na_V_1.5 (IH)					House et al., 2010
		HT-29		Na_V_1.5 (IH)		Current	Na_V_1.5-dependent invasion (shown with 30 μM TTX or siRNA)		House et al., 2010
		SW620		Na_V_1.5 (IH)		Current	Na_V_1.5-dependent invasion (shown with 30 μM TTX or siRNA)		House et al., 2010
		SW480		Na_V_1.5 (IH)		Current	Na_V_1.5-dependent invasion (shown with 30 μM TTX or siRNA)		House et al., 2010
	Non-cancer	(Biopsies of normal colon)		Na_V_1.5 faint staining (IH)					House et al., 2010
Ovary	Cancer	Anglne	Na_V_1.1 Na_V_1.2 Na_V_1.3 Na_V_1.4 Na_V_1.5 Na_V_1.6 Na_V_1.7 Na_V_1.8 Na_V_1.9	Na_V_1.5 (IH)			—		Gao et al., 2010
		Caov-3	Na_V_1.1 Na_V_1.2 Na_V_1.3 Na_V_1.4 Na_V_1.5 Na_V_1.6 Na_V_1.7 Na_V_1.8 Na_V_1.9	Na_V_1.5 (IH)			TTX-R migration and invasion		Gao et al., 2010
		SKOV-3	Na_V_1.1 Na_V_1.2 Na_V_1.3 Na_V_1.4 Na_V_1.5 Na_V_1.6 Na_V_1.7 Na_V_1.8 Na_V_1.9	Na_V_1.5 (IH)			TTX-R migration and invasion		Gao et al., 2010
	Non-cancer	(Biopsies of normal ovary)	Na_V_1.1 Na_V_1.2 Na_V_1.3 Na_V_1.4 Na_V_1.5 Na_V_1.6 Na_V_1.7 Na_V_1.8 Na_V_1.9	Na_V_1.5 (IH)				Yes (RT-PCR)	Gao et al., 2010
		Primary-cultured luteinized cells of the granulosa	Na_V_ (type not indicated) (RT-PCR)	Na_V_α protein (type non identified) detected with panNa_V_α antibody (WB, IH)	6.8 nM	TTX-S current	Physiological luteolysis of normal corpus luteum cells of the ovary		Bulling et al., 1999, 2000

*IH, immune-histochemical staining; nNa_V_, neonatal isoform; ?, not reported; —, not found*.

### Pore-forming Na_*v*_α proteins in cancer cells

The first descriptions of voltage-dependent sodium currents, and therefore of the functional expression of Na_V_α proteins at the plasma membrane of cancer cells, and the hypotheses that they could participate in the oncogenic process came in the late 1980's. In these pioneer works voltage-gated sodium currents were initially recorded in human leukemia cells (Yamashita et al., 1987; Lee et al., 1988), then in human small-cell lung cancer cells (Pancrazio et al., 1989). Initial works performed in leukemia cells were aimed at studying the participation of plasma membrane conductances in the acquisition of the multidrug resistance (MDR) phenotype that is a major problem leading to loss of efficacy of cancer chemotherapy, often associated with a gain of aggressiveness. In the first of this two studies, two human leukemia cell lines were characterized at the electrophysiological level, the drug-sensitive K562 and the adriamycin-resistant cell line K562/ADM that was selected on the basis of its resistance to multiple drugs including anthracyclines and *Vinca* alkaloids. In contrast to K652 cells which only showed K^+^ outward currents, most of the K562/ADM cells also exhibited fast inward voltage-gated sodium currents that were fully inhibited by 1 μM TTX. Moreover, sodium currents were no longer observed in revertant cells coming from the K562/ADM cell line having a reduced MDR phenotype after being grown for 6 months in absence of adriamycin. While TTX had no effect on cell growth or on vincristine uptake in K562/ADM cells, these authors concluded that TTX-sensitive Na_v_ was associated with, albeit not directly involved in, drug resistance (Yamashita et al., 1987). In the second study, the drug-sensitive human T-cell leukemia cell line CCRF-CEM was compared to its MDR variant, vinblastine-resistant CEM/VLB_100_. In both cell lines, an identical TTX-sensitive I_Na_ was found, suggesting a similar level of expression of Na_V_α proteins. Furthermore, 1 μM TTX did not affect the proliferation of CCRF-CEM and CEM/VLB_100_ cells, while it fully inhibited I_Na_, and the role of this conductance remained unidentified (Lee et al., 1988). The channels responsible for these TTX-sensitive sodium currents werenot characterized at that time, and it was not possible to know whether only one or multiple Na_V_α were expressed, and whether these were the same Na_V_α proteins in all leukemia cell types.

Later on, I_Na_ were recorded in human H146, H69, and H128 small-cell lung cancer cell lines. These currents were most probably due to TTX-sensitive channels since they fully inhibited by 5 μM TTX, and while their biological role was not assessed, they were proposed to participate to a “neuroendocrine-like” tumor cell phenotype (Pancrazio et al., 1989). This initial study, along with subsequent works conducted in different labs, were probably at the origin of the hypothesis defended by Djamgoz and his team, discussing for a “neuroscience” approach in oncology (Onganer et al., 2005). During the following two decades, between the 1990's and the 2010's, the idea came that cancer cells, originating from different non-excitable tissues, acquired membrane excitability that would be associated with cancer progression. However, the existence of such action potentials, being either spontaneous or triggered, in carcinoma cells is still questioned. This “excitability” hypothesis is discussed hereafter, and while it is not the one that we are supporting, it could have appeared a tempting proposal with regard to the well-known role of Na_V_ in excitable cells. Djamgoz and researchers from his team brought an important contribution to the field of Na_V_ channels in cancer. Many of their studies clearly showed the expression of functional Na_V_α proteins in cancer cells, as in rat (Grimes et al., 1995; Grimes and Djamgoz, 1998) and human (Laniado et al., 1997) prostate cancer cells, T-cell leukemia (Fraser et al., 2004), human small-cell lung cancer cells (Onganer and Djamgoz, 2005), and more recently proposed it as a therapeutic target in ovarian cancer cells (Frede et al., 2013). From the very beginning, the presence of I_Na_ was associated with the enhanced migration and the invasive phenotype of cancer cells (Smith et al., 1998; Djamgoz et al., 2001; Fraser et al., 2003), while the cellular mechanisms proposed to be involved remained elusive until recent studies (Brisson et al., 2011, 2013; Yang et al., 2012a).

In their first study, they recorded I_Na_ in the highly invasive and metastatic Mat-Ly-Lu, but not in the weakly invasive AT-2, rat prostate cancer cells. Sodium currents were fully inhibited by 1 μM TTX, suggesting the expression of TTX-sensitive Na_V_α proteins at the plasma membrane of prostate cancer cells (Grimes et al., 1995). In Mat-Ly-Lu cell line, TTX was not interfering with cancer cell proliferation but reduced by about 33% cancer cell invasiveness through an invasion chamber coated with an extracellular matrix composed of Matrigel™. In contrast, TTX had no effect on the invasiveness of AT-2 cells. Later on, similar results were obtained in human prostate cancer cells: TTX-sensitive I_Na_ was recorded in highly invasive PC-3 cells and its pharmacological inhibition significantly reduced cell invasiveness, while no current was observed in the weakly invasive LNCaP cells (Laniado et al., 1997). In these two studies performed with prostate cancer cell lines, while I_Na_ was only recorded in highly invasive cells PC-3 or Mat-Ly-Lu, it appeared that the expression of Na_V_α seemed to be restricted to a subpopulation of cells since I_Na_ was not recorded in all investigated cells. This particularity was further studied in different human and rat prostate cancer cell lines by flow cytometry using a fluorescein-labeled polyclonal Pan-Nav antibody, and the authors found a positive correlation between the proportion of cells expressing Na_V_α at the plasma membrane and the invasive capacity (Smith et al., 1998).

Following these studies, the Na_V_α protein responsible for TTX-sensitive currents in prostate cancer cells was investigated, and from this attempt came confusing results that however demonstrate that the Na_V_α isoform expressed *per se* might not be critical for the biological effect in cancer cells. Indeed, the study from Diss and collaborators indicated that the mRNA encoding the full-length skeletal muscle type 1 (SkM1), now identified as being Na_V_1.4, was present in the Mat-Ly-Lu cell line and also expressed in the PC-3 cells. *In situ* hybridization suggested that Na_V_1.4 transcripts were expressed in both Mat-Ly-Lu and AT-2 cells but with a markedly different expression level since highly invasive Mat-Ly-Lu and PC-3 cells exhibited much higher levels (Diss et al., 1998). These results indicated (1) that Na_V_1.4 mRNA could be expressed in other cells than skeletal muscle cells, and (2) that the expression level of Na_V_1.4 mRNA seemed to be associated with the invasive capacities of cancer cells, known to express functional Na_V_α. Here is the origin of the confusion: while authors of this study never concluded that Na_V_1.4 was responsible for I_Na_ in highly invasive prostate cancer cells, the inferred association might have been formulated in the mind of many researchers.

After this study, Bennett and collaborators compared the expression of Pan-Na_V_α proteins by immunoblotting in the weakly invasive LNCaP human prostate cancer cells, and two derivative cell lines, C4 and C4-2, which present increasing invasive properties. They found a positive correlation between Na_V_α expression and the increased invasiveness of cancer cell lines, and that the use of TTX (1 μM) reduced the matrix invasion by these three cell lines to the same level (Bennett et al., 2004). Also of interest is the fact that the transient overexpression of Na_V_1.4 proteins in cancer cells increased their invasiveness. These results perfectly fitted with the initial study from Diss et al. (1998) and it was proposed that Na_V_1.4 expression was sufficient to increase prostate cancer cell invasiveness. However, by using degenerate primer screening and semi-quantitative PCR, it was later found that PC-3 and Mat-Ly-Lu cells express mRNA for Na_V_1.4, but also for Na_V_1.1 and Na_V_1.2 channels and that the predominant isoform was the TTX-sensitive Na_V_1.7. This channel was likely to be responsible for I_Na_ in prostate cancer cells (Diss et al., 2001). Na_V_1.7 was found to be overexpressed, in mRNA and in proteins, in prostate cancer biopsies compared to non-cancer prostate tissues, and it was even proposed to serve as a diagnosis marker for prostate cancer (Diss et al., 2005). These results demonstrated that, even if Na_V_1.7 might be the channel primarily overexpressed and involved in the invasive properties of prostate cancer cells, the mechanisms responsible for enhancing cancer cell invasiveness can be reproduced by the overexpression of another Na_V_α. This is very important because it suggests that the activity of Na_V_α, i.e., I_Na_, might be more important than the molecular identity of the channel, and that broad spectrum inhibitors of Na_V_ rather than some being very specific to isoforms, could be used to reduce cancer cell invasiveness.

In breast cancers, it appeared that the most expressed was **Na**_V_**1.5** (Fraser et al., 2005; Gillet et al., 2009). In the strongly metastatic MDA-MB-231 human breast cancer cell line, all tested cells express voltage-gated sodium currents. These currents were initially found to be TTX-resistant, with an IC_50_ of approximately 2 μM and required 30 μM TTX to be fully inhibited. This current was not recorded in weakly invasive MCF-7 or MDA-MB-468 breast cancer cells, nor in non-cancerous MCF-10A epithelial mammary cells (Roger et al., 2003; Brisson et al., 2011). Its pharmacological inhibition, using the pore-blocker TTX (Roger et al., 2003; Gillet et al., 2009; Brisson et al., 2011), the antiarrhythmic ranolazine (Driffort et al., 2014), or the antiepileptic phenytoin (Yang et al., 2012a) had no effect on MDA-MB-231 cell proliferation, nor on cell migration through a non-coated filter with 8-μm-diameter pores, but reduced by 30 to 40% the invasion through the same filters coated with a film of Matrigel™ as an extracellular matrix. While MDA-MB-231 cells express mRNA for multiple Na_V_α such as Na_V_1.2, Na_V_1.4, and Na_V_1.8 at low level, and Na_V_1.5, Na_V_1.6, and Na_V_1.7 at high levels (Jude et al., 2006), only Na_V_1.5 seemed active at the plasma membrane. Targeting *SCN5A* expression using specific siRNA completely abolished the presence of I_Na_ and had the same effect as TTX for reducing cancer cell invasiveness. Conversely, increasing the activity of Na_V_1.5 channels using veratridine increased cancer cell invasiveness (Gillet et al., 2009). This channel was found to be expressed as a neonatal splice variant, called nNa_V_1.5, showing seven amino acids substitutions in the second extracellular loop (E2) of the first domain (DI) of the channel compared to the adult isoform (aNa_V_1.5) (Fraser et al., 2005; Brackenbury et al., 2007). This molecular domain is critical for Na_V_ pharmacology and neonatal isoforms display functional and pharmacological specificities, such as the sensitivity to some other drugs such as verapamil and diltiazem, but not nifedipine (Roger et al., 2004b). Na_V_1.5 was found to be overexpressed in breast cancer biopsies compared to non-cancerous biopsies, associated with lymph node invasion, metastatic relapse and a decreased survival in patients (Fraser et al., 2005; Yang et al., 2012a).

In a human leukemia model, it was found by patch-clamp recordings that a subpopulation of approximately 10% of Jurkat T cells was expressing TTX-resistant sodium currents, with an IC_50_ around 1 μM. The expression of Na_V_α proteins was confirmed by western blotting using a Pan-Na_V_ antibody. Semi-quantitative PCR analyses revealed the expression of mRNAs for multiple Na_V_α such as Na_V_1.7 (TTX-sensitive) and Na_V_1.9 (TTX-resistant) at low levels, and Na_V_1.5 (TTX-resistant) and Na_V_1.6 (TTX-sensitive) at much higher levels. Correlatively to the pharmacology of the sodium current recorded it was proposed that the channel most likely to be functional was **Na**_V_**1.5**. Inhibiting the current with 10 μM TTX reduced Jurkat cell invasiveness through Matrigel™-coated inserts by more than 90% (Fraser et al., 2004).

In human non-small-cell lung cancer cell lines it was found that the strongly metastatic H23, H460 and Calu-1 cancer cells possessed I_Na_ while the weakly invasive A549 and the non-cancer lung epithelial cells NL-20 and BEAS-2B did not. The presence of I_Na_ was associated with a significant increase in the intracellular concentration of Na^+^ in cancer cells (15.3 ± 2.2 mM) compared to non-cancer cells (7.8 ± 1.3 mM). Again, the inhibition of these sodium currents, using different concentrations of TTX had no effect on cancer cell proliferation or on cell migration, but reduced cancer cell invasiveness through an extracellular matrix by about 50% (Roger et al., 2007). However, the Na_V_α channels likely to be responsible for this activity in the three studied cell lines might be different. Indeed, while the TTX sensitivity of sodium currents recorded in H23 and H460 cells was in the nanomolar range, and therefore considered as being TTX-sensitive, in Calu-1 cells it appeared that there were two populations of currents, one being TTX-sensitive and one being TTX-resistant. The use of TTX at different concentrations to partially inhibit TTX-S (5 nM), fully inhibit TTX-S and partially TTX-R (1 μM) or fully inhibit all channels (30 μM) produced dose-dependent reductions of Calu-1 invasiveness. It was concluded that both types of Na_V_α, TTX-S and TTX-R, were equally involved in the invasive properties, and that only the activity was important. Surprisingly, semi-quantitative RT-PCR experiments indicated that all the studied cell lines, cancerous and non-cancerous, expressed mRNA for Na_V_α, and especially for the TTX-S Na_V_1.6 and Na_V_1.7. In the case of H23 and H460 cells, **Na**_V_**1.7** mRNA was the most abundant and was proposed to be responsible for I_Na_ (Roger et al., 2007). This was later confirmed in H460 cancer cells, where using specific siRNA targeting Na_V_1.7 to inhibit its expression led to a decrease in invasiveness, and in weakly invasive A549 cells where transfection to force the expression of the channel was responsible for an increased invasiveness (Campbell et al., 2013). Interestingly, H23 and H460 cells also expressed mRNA for Na_V_1.5, but the TTX sensitivity of I_Na_suggested that it was not functionally expressed. Calu-1 cells expressed mRNA for all Na_V_α, except for Na_V_1.4. The particular pharmacology of the current along with kinetics of activation and inactivation indicated the TTX-R channel likely to be functional at the plasma membrane might be **Na**_V_**1.5**, while the TTX-S current could be due to Na_V_1.1, Na_V_1.3, Na_V_1.6, and/or even Na_V_1.7 activity (Roger et al., 2007). Of interest is the fact that poorly aggressive A549 cells expressed Na_V_α proteins in intracellular compartments but not at the plasma membrane, and that TTX (30 μM) applied extracellularly (but possibly endocytosed), induced no effect on cell invasiveness. This suggests that Na_V_ activity at the plasma membrane is critical for inducing matrix invasion. In this study, it was surprising to notice that non-cancer cells, NL-20 and BEAS-2B, also expressed mRNA for Na_V_1.6, Na_V_1.7, plus Na_V_1.5 for the latter, had Na_V_α proteins as observed in western blotting experiments, but that these proteins seemed to be incorrectly matured and not addressed at the plasma membrane (Roger et al., 2007). These cells are non-cancer cells and non-tumorigenic when injected to immunodepressed mice. However, they cannot be considered as being normal, since they have been immortalized and might bear some mutations. Therefore, it is tempting to speculate that functional expression of Na_V_α at the plasma membrane is a feature of highly invasive cancer cells with multiple mutations accumulated, while the mere expression of Na_V_α mRNA/protein might be deregulated at the earlier stages of the carcinogenic process.

In human colon cancer cell lines SW620, SW480, and HT29, the expression of mRNA was identified for several Na_V_α, with the most abundant transcripts being those encoding the TTX-R **Na**_V_**1.5**. In these three cell lines, the presence of the Na_V_1.5 proteins at the plasma membrane was confirmed by immunocytochemistry, and their functionality was identified by measuring TTX-R I_Na_ (partially inhibited by 10 μM TTX) in patch-clamp recordings (House et al., 2010). Here again, the use of TTX (30 μM in order to inhibit >70% of sodium current) or siRNA specifically targeting Na_V_1.5 expression, induced a significant reduction of cancer cell invasiveness, by approximately 50%, compared with untreated or non-sense control siRNA-treated cells. SW620 cells were shown to express both adult and neonatal Na_V_1.5 splice variants. Increasing the activity of the channels with veratridine (10 μM) promoted cancer cell invasiveness, while inhibiting them using the local anesthetics lidocaine (10 μM) or ropivacaine (2.5 μM) had opposite effects (Baptista-Hon et al., 2014). The presence of Na_V_1.5 proteins at the plasma membrane of the luminal surface of cancer cells was confirmed by immunohistochemistry in a panel of human colon cancer specimens. By comparison, normal-matched control samples showed very faint or no staining. This suggested that functional expression of Na_V_1.5 might be repressed in normal tissues but that they are aberrantly expressed in colon cancer cells (House et al., 2010). In the same study, authors also used a powerful bioinformatics approach combining transcriptomic analyses and invasion experiments in order to identify networks of invasion-related genes, the expression of which could be regulated by, or related to, Na_V_1.5 expression in colon cancer cells (House et al., 2010).

A recent study assessed the mRNA expression of Na_V_1.6 channels (*SCN8A* gene) using real-time quantitative PCR in colorectal carcinomas (*n* = 64 patients). A significantly lower expression of *SCN8A* was found in colorectal tumor tissues compared to paired tumor-surrounding non-cancerous tissues, and in patients below the age of 45 compared to older patients. Of all the other patient parameters evaluated as a function of *SCN8A* expression, such as gender, tumor grade, tumor location (cecum vs. right colon) and histopathological classification, there was no significant relationship (Igci et al., 2015). However, the significance of such findings remains unclear since the expression of other Na_V_α was not assessed, it is not indicated whether the transcript evaluated was an adult or a neonatal variant. Furthermore, Na_V_1.6 protein expression and activity were not evaluated.

The team of Gomora demonstrated the functional expression of TTX-sensitive Na_V_α in primary cultures established from human cervical cancer biopsies. By using RT-PCR and specific primers they looked for the expression of the TTX-sensitive Na_V_1.2, Na_V_1.4, Na_V_1.6, and Na_V_1.7 channels which were all amplified in cervical cancers while only mRNA for Na_V_1.4 were identified in normal cervix tissues (Diaz et al., 2007). Another study from the same group indicated that mRNA levels of Na_V_1.6 were approximately 40-fold higher in cervical cancer biopsies than in non-cancerous cervical tissues, and that Na_V_1.7 mRNA expression was also increased approximately 20-fold in cancer tissues. Moreover, immunohistochemistry experiments demonstrated that both Na_V_1.6 and Na_V_1.7 proteins were significantly more expressed in cancer biopsies and in primary culture of cancer cells compared to non-cancer tissues, and widely distributed in both cytosolic and plasma membrane compartments. The activity of **Na**_V_**1.6** channels in the plasma membrane of cervical cancer cells was confirmed using the Na_V_1.6-specific toxin Cn2 (1 μM) which blocked ~50% of the sodium current in patch-clamp experiments, and reduced by ~20% cell invasiveness of primary-cultured cervical cancer cells, without affecting cell proliferation or migration. TTX (1 μM) had a similar effect on cancer cell invasiveness (Hernandez-Plata et al., 2012).

The expression of Na_V_α was also assessed in human ovarian cancer cell lines and in ovarian cancer biopsies, which were compared with normal ovary samples. The relative mRNA expression, analyzed by RT-PCR, was found higher for Na_V_1.1, Na_V_1.3, Na_V_1.4, and Na_V_1.5 in ovarian cancers cells compared with normal ovarian tissues, and the mRNA expression of Na_V_1.2, Na_V_1.4, Na_V_1.5, and Nav1.7 was also significantly increased in highly invasive ovarian cancer cells Caov-3 and SKOV-3, compared with the weakly invasive Anglne ovarian cancer cells, while the expression of mRNA for other Na_V_α was not significantly different. The expression of mRNA and proteins for **Na**_V_**1.5** seemed to be increased in cancer tissues and seemed to be correlated with the grade and metastasis status of ovarian cancers. *In vitro*, the treatment of Caov-3 and SKOV-3 cells with 30 μM TTX reduced cell invasiveness by 50–60%, while 1 μM TTX had no effect. The authors suggested that the abnormal expression of Na_V_1.5 could be associated with the metastatic process in human ovarian cancer and could be used as a therapeutic target in ovarian cancer treatment (Gao et al., 2010).

From all these studies it appears that several Na_V_α such as **Na**_V_**1.5, Na**_V_**1.6, and Na**_V_**1.7** have been unequivocally shown to be abnormally expressed and functional in different cancer types. The reason why these particular channels are overexpressed in cancers, and the transcriptional deregulations responsible for this are not known and might be tissue-specific. However, as previously suggested, the molecular identity of the channel might not be very important as long as a sodium current exists at the plasma membrane. While these currents could be recorded in experimental conditions ensuring for a large current, i.e., using the patch-clamp technique, and performing a highly depolarized voltage step (such as −10 or 0 mV) from a very hyperpolarized membrane potential (−100 or −120 mV), a question rapidly appeared in this context: how could these channels be functional in cancer cells for which the membrane potential is rather depolarized compared to excitable cells (Yang and Brackenbury, 2013) and generally comprised between −40 and −30 mV (Roger et al., 2003, 2004a,c, 2007; Gillet et al., 2009, 2011)? Indeed, the relatively depolarized membrane potential reported in cancer cells would significantly decrease the availability of Na_V_ and would preclude for action potential initiation.

When looking at the activation-voltage and inactivation-voltage relationships of these channels, it was demonstrated that a window of voltage always existed, in which channels can be activated and not fully activated, and that the mean membrane potential of the studied cancer cells was within this window of voltage (Roger et al., 2003, 2004a, 2007; Fraser et al., 2004; Diaz et al., 2007; Gillet et al., 2009; Campbell et al., 2013). This suggested that Na_V_α could be functional, through a window current that could be considered as being a “basal activation” of channels. This small but persistent entry of sodium into cancer cells is likely to be responsible for the increase in intracellular sodium content that has been demonstrated a long time ago in cancer tissues compared to non-cancer tissues (Cameron et al., 1980). The relationships between the increased intracellular concentration of Na^+^ and the enhancement of cancer cell invasiveness are still very unclear and will be discussed in the following part of this review.

Even though mechanistic details on the participation of Na_V_ to the invasive properties of cancer cells remain incomplete, it became very promising to search for ways to decrease the expression of such channels in cancer cells, or alternatively to inhibit their activity in order to reduce metastatic colonization of organs. Recent studies clearly demonstrated that the pharmacological inhibition of Na_V_α was very potent to prevent the development of metastases in rodent models, such as the use of TTX (Yildirim et al., 2012), phenytoin (Nelson et al., 2015), or ranolazine (Driffort et al., 2014). Another way to regulate the aggressivity of breast cancer cells could be the transcriptional inhibition of Na_V_1.5 expression in breast cancer cells by n-3 polyunsaturated long chain fatty acids coming from the diet, such as docosahexaenoic acid (C22:6n-3) (Isbilen et al., 2006; Gillet et al., 2011), that was recently identified as being mediated by the lipid-sensitive transcription factor Peroxisome Proliferator Activated Receptor (PPAR)-β (Wannous et al., 2015).

### Non pore-forming Na_v_β proteins in cancer cells

The participation of non-pore-forming Na_V_β in oncogenic processes was not studied as much as that of Na_V_α, but it becomes more and more obvious that they could play important roles during carcinogenesis and cancer progression toward high metastatic grades. The most studied Na_V_β protein in cancer is β1, which results from the expression of the *SCN1B* gene. Initial studies indicated that β1 expression was high in poorly invasive MCF-7 breast cancer cells and lower in highly invasive MDA-MB-231 breast cancer cells *in vitro*. However, the participation of β1 in the invasive phenotype was not very clear. In weakly invasive MCF-7 cells, which do not express functional Na_V_α, the transfection of siRNA targeting β1 reduced cell adhesion by 35%, and increased cell migration by about 120%. Its overexpression in highly invasive MDA-MB-231 cells, endogenously expressing functional Na_V_1.5, increased cell process length and adhesion, and reduced lateral motility and cell proliferation (Chioni et al., 2009). Surprisingly, the overexpression of β1 increased the activity of the pro-invasive sodium channel, which, expectedly, should have increased cell invasiveness. These results suggested that β1 expression could prevent cancer cell invasion. In a recent study, it was nicely demonstrated that *SCN1B* mRNA and β1 proteins were up-regulated in breast cancer biopsies, compared with non-cancer breast samples. MDA-MB-231 overexpressing β1 were significantly more invasive than untransfected control cells, through both Na_V_α-dependent and Na_V_α-independent mechanisms, and generated bigger primary tumors, more and bigger metastatic foci, when implanted into the mammary fat pads of immunosuppressed *Rag2*^−∕−^ and *Il2rg*^−∕−^ mice (Nelson et al., 2014). The overexpression of β1 seemed to increase primary tumor vascularization and to reduce cancer cell apoptosis. β1-over expressing cancer cells also displayed an elongated morphology, with the formation of cellular process outgrowths requiring the activity of Fyn kinase, and Na_V_1.5 current (Nelson et al., 2014). Consistently with this, *SCN1B* mRNA was found to be more abundant in highly invasive than in weakly invasive prostate cancer cell lines (Diss et al., 2008).

Not many studies have been focused on the potential participation of the β2 subunit in cancer cell properties. This subunit is similar to neural CAMs and is known to form both homotypic and heterotypic interactions with several CAMs, among which the β1 subunit (Isom et al., 1995; Malhotra et al., 2000). Subunit β2 interacts with glycoproteins from the ECM such as Tenascin-C and Tenascin-R (Srinivasan et al., 1998; Xiao et al., 1999) which are important factors regulating physiological cell proliferation and migration during embryogenesis, particularly in neurogenic areas (Midwood et al., 2011; Anlar and Gunel-Ozcan, 2012), but also cancer cell proliferation and migration (Brellier and Chiquet-Ehrismann, 2012). Therefore, it was initially postulated that the increased expression of β2 in cancer cells could promote cancer progression. It was initially observed that β2 was overexpressed in the metastatic prostate cancer cell subline C4-2B compared to the weakly metastatic mother cell line LNCaP from which it is derived (Jansson et al., 2012). Furthermore, the overexpression of the β2 protein, bearing a GFP tag at the intracellular C-terminus, induced a change in LNCaP cell morphology with the acquisition of a bipolar and elongated phenotype. This was also accompanied by an increase in cell adhesion to vitronectin and Matrigel™, but not to growth factor-reduced Matrigel™ or to fibronectin, and the increase in cell migration and in Matrigel™ invasion, with no significant effect on cell proliferation. In contrast to what could have been initially expected, the overexpression of β2 in LNCaP reduced the growth of the xenogenic tumor generated by subcutaneous injection of cells in balb/c nude mice (Jansson et al., 2012). Later on, the same group demonstrated in organotypic spinal cord co-cultures, that LNCaP prostate cancer cells overexpressing β2 displayed an enhanced association with nerve axons (Jansson et al., 2014). It also increased cancer cell migration, invasion and proliferation on laminin, suggesting that the overexpression of β2 in prostate cancer cells may be involved in metastatic perineural invasion (Jansson et al., 2014).

While the role of β3 in cancer cell biology is uncertain, it is of interest to notice that two missense mutations have been identified in the *SCN3B* gene in high grade metastatic colorectal cancer biopsies (Sjoblom et al., 2006), and that non-mutated *SCN3B* gene was proposed to induce apoptosis through a p53-dependent signaling pathway in cancer cells after DNA damages (Adachi et al., 2004). The *SCN3B* gene is not expressed at all in highly invasive MDA-MB-231 breast cancer cells (Gillet et al., 2009) and is found at very low level in non-small-cell lung cancer cells (Roger et al., 2007; Campbell et al., 2013). In contrast, *SCN3B* expression was increased in cervical cancer biopsies when compared to non-cancer samples (Hernandez-Plata et al., 2012). In the same study, *SCN2B* and *SCN4B* expression was decreased in cervical cancer biopsies (Hernandez-Plata et al., 2012). *SCN4B* expression appeared to be very low in invasive breast cancer cells (Gillet et al., 2009), and decreased in invasive compared to non-invasive prostate cancer cells (Diss et al., 2008).

These results suggest that Na_V_β may be involved in cancer cell biology. However, other studies are necessary to clearly establish their respective and complementary roles in cancer cell properties such as cell proliferation and resistance to apoptosis, cell adhesion, migration and extracellular matrix invasion. It will also be important to determine whether these properties are dependent, or not, on the expression of Na_V_α.

## Molecular mechanisms for the regulation of cancer cell biology by voltage-gated sodium channels and comparison with non-excitable and non-cancer cells

Important questions still remain: how can Na_V_α activity, through a persistent entry of Na^+^, be responsible for the increased invasiveness observed in cells that do not trigger action potentials? And what are the signaling pathways involved? It is widely known that many intracellular signaling pathways crucially depend on Ca^2+^ ions for which a myriad of Ca^2+^-sensitive proteins exist. Could it be the same for Na^+^ ions? The way by which Na_V_ activity controls cancer cell invasiveness remained unknown for long and is still unclear despite the recent advances that have been done and will be discussed later on. In order to understand a possible signaling pathway that could be under the control of Na_V_ activity, but independent of action potential generation, we will give a rapid overview of the current knowledge on the physiological participation of Na_V_ channels in non-excitable cells. We will compare this to Na_V_-dependent mechanisms that have been identified in cancer cells.

### Normal expression and non-excitable roles of Na_v_ in non-cancer cells

**Figure 1:**
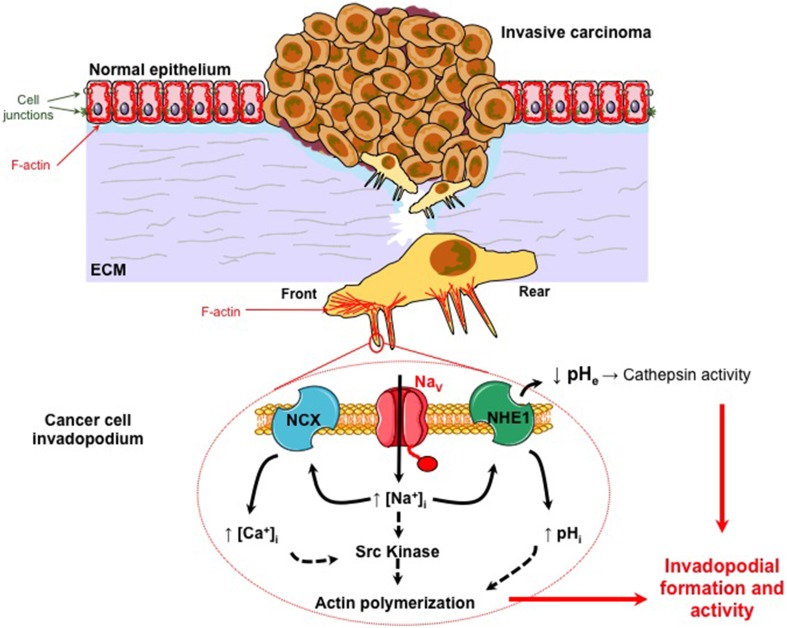
**Na_V_α promotes carcinoma cell invasiveness and metastatic progression**. Normal epithelial cells are morphologically and functionally polarized (apico-basal polarization). They are tightly connected together by different cell junctions such as tight junctions, adherens junction, gap junctions, and desmosomes. They are associated to the basement membrane by hemidesmosomes composed of integrins. They are maintaining a particular organization of the cytoskeleton. Particularly, actin filaments (F-actin) form a cortical network under the plasma membrane that is determinant in keeping this polarized morphology. During the carcinogenetic process, cancer cells that survived to environmental constraints, and have been selected, have acquired multiple mutations conferring proliferative and immortality advantages. This process is responsible for the development of a primary *in situ* tumor that becomes invasive when some aggressive cancer cells gain the ability to degrade and migrate through the extracellular matrix (ECM). This second step is critical for invasive cancer cells to reach the blood circulation and eventually to colonize and form secondary tumors (metastases) in distant organs. Migrative cancer cells have lost their intercellular junctions, their apico-basal polarization and now display a front-rear polarization. The proteolysis of the ECM by cancer cells is dependent on the formation and activity of protrusive structures, enriched in F-actin, called invadopodia. All these changes in cell phenotype and behavior are obtained through the process called Epithelial-to-Mesenchymal Transition (EMT). Na_V_α proteins are abnormally expressed in highly invasive cancer cells, and are found in invadopodia (see magnification). They are co-localized with Na^+^-H^+^ exchangers type 1 (NHE-1) for which they enhance the protons extrusion activity. This leads to a peri–invadopodial acidification favorable to the activity of acidic cysteine cathepsins, released by cancer cells, and consequently to the ECM degradation. Na_V_α proteins are also proposed to increase the intracellular Ca^2+^ concentration through the participation of Na^+^-Ca^2+^ exchangers (NCX). Na_V_α activity, through a persistent window current, sustains Src kinase activity, the phosphorylation (Y421) of the actin nucleation-promoting factor cortactin, and the polymerization of actin filaments. Altogether, this suggests that Na_V_α activity in cancer cells enhances both the formation and the ECM degradative activity of invadopodia.

Sodium channels, especially Na_V_α proteins, have been found in many cell types not described for the generation of action potentials, such as in epithelial gastric cells (Wu et al., 2006) in which Na_V_1.5 participates to the control of cell proliferation, in astrocytes in which the same channel participates to cell migration (Pappalardo et al., 2014), in macrophages and microglial cells in which Na_V_1.6 channel regulates the phagocytic capacity (Craner et al., 2005; Black and Waxman, 2012) and Na_V_1.5 participates to the endosomal acidification (Carrithers et al., 2007, 2009), or in dendritic cells in which Na_V_1.7 maintains the membrane potential and regulates the activation and chemokine-induced migration (Kis-Toth et al., 2011). Na_V_α have been characterized to be expressed both at the plasma membrane and in intracellular compartments of many other cell types, and the hypothesis has emerged that they could physiologically play non-excitable roles (for a recent review, see Black and Waxman, 2013). It is very interesting to note the recurrent participation of Na_V_ channels in normal cell migration/invasiveness, phagocytosis, endosomal acidification, and podosomal activities, that are cellular properties over-activated in cancer cells and often associated with aggressiveness (Brisson et al., 2012) (see Figure 1).

Non-excitable roles of Na_V_ channels in cell migration and proliferation during embryogenesis and tissue repair could be relevant to explain similar phenomena in cancer cells. They will be briefly described thereafter.

#### Sodium channels and the regulation of Ca^2+^ concentration in smooth muscle and other cells

Sodium currents have been measured in many primary-cultured smooth muscle cells from human pulmonary arteries (James et al., 1995), human coronary arteries (Quignard et al., 1997), mesenteric arteries (Berra-Romani et al., 2005), or from aortic arteries (Cox et al., 1998). These smooth muscles cells are not excitable (if one considers excitability as the capacity to produce action potentials) and at least one of their primary roles has been unraveled in rat aorta (Fort et al., 2009). Large arteries such as the aorta do not show action potentials but rather display small variations in their membrane potential. A depolarization as small as 10 mV has been shown to increase the vascular tone. This was prevented by the use of 1 μM TTX. The hypothesis put forward by the authors was that the increase in the intracellular sodium concentration in turn induced an entry of calcium, through the sodium-calcium exchanger (NCX), which was responsible for the increase in cell contraction (Fort et al., 2009). It has never been tested whether Na_V_ activity could also regulate smooth muscle cell proliferation in physiological, or even in pathophysiological conditions such as in pulmonary hypertension. Nevertheless, this mechanism of action resembles that found for Na_V_ channels expressed in NG2 oligodendrocyte progenitor cells. In these cells, the stimulation of GABA_A_ receptors induced a membrane depolarization activating Na_V_ channels. These channels were responsible for a non-inactivating I_Na_, leading to an increased intracellular Ca^2+^ concentration and an enhanced cell migration due to the reverse mode functioning of NCX-1, (Tong et al., 2009). A similar mode of action was recently published for astrocytes migration in astrogliosis (Pappalardo et al., 2014).

#### Sodium channels in cardiac and in neuron cells during embryogenesis

It is well-known that the sodium current (Weidmann, 1961) due to the activity of Na_V_1.5 is responsible for the excitability of cardiac cells. However, a careful examination of the expression of Na_V_ channels during cardiac development suggests that Na_V_1.5 is also involved in the development of the heart independently of the generation of action potentials. Indeed, it has been reported in mice that a homozygous deletion of the cardiac sodium channel gene *SCN5A* resulted in the death of embryos at the age of 10.5 days, before a large sodium current can be seen (Davies et al., 1996; Papadatos et al., 2002). Embryos had small ventricles, but had intact endocardial tissues and vasculature (Papadatos et al., 2002). Similar observations have been seen in zebrafish embryos (Chopra et al., 2010). These authors observed that the heart tube of zebrafish lacking the ortholog gene *SCN5Lab* (one of the two orthologs of the *SCN5A* gene) had less cardiomyocytes than the wild type zebrafish 24 h after fertilization, and that the embryos died before 5 days post-fertilization. Later on, Bennett and collaborators demonstrated that the Na_V_ channel expressed by the *SCN5Lab* gene is required for the specification of the pre-cardiac mesoderm, and for the proliferation and growth of the developing heart (Bennett et al., 2013). The precise mechanism of action is not yet understood but these authors underlined that, as an integral component of cardiomyocytes, Na_V_ could serve non-electrogenic roles by interacting with many partner proteins.

Interestingly, Na_V_ channels appear to have critical non-excitable roles during embryogenesis. This was also identified during neuronal embryogenesis in mouse neocortex. Indeed, long before synaptogenesis, the early expression and function of Na_V_ have been involved in neuronal development and in the colonization of the neocortex by pioneer neurons originating from the preplate. It was showed that preplate neurons of the mouse neocortex express functional Na_V_1.3 as early as day 12 of embryonic development, and that the activity of the channel was not related to cell excitability (Albrieux et al., 2004). This channel was proposed to increase the intracellular concentration of Ca^2+^ through the participation of the NCX exchanger, thus triggering Ca^2+^-dependent signaling in the preplate neurons, leading to glutamate secretion and the development of the neocortex before functional synapses are formed (Platel et al., 2005).

#### Sodium channels in tissue regeneration

A very exciting study came from the group of Levin, working on the regeneration of an entire organ in *Xenopus laevis* larvae (Tseng et al., 2010). It is known that during embryogenesis, amphibians can regenerate and restore amputated, complex organs, containing different cell types such as those constituting muscles, peripheral nerves, vasculature, skin, etc. The authors of this study demonstrated that Na_V_1.2 expression and activity was critical for tail regeneration. The expression of *SCN2A* gene was up-regulated 18 h after tail amputation. Such a transcriptional regulation of *SCN2A* seemed to occur after the membrane hyperpolarization mediated by the activity of the H^+^ pump V-ATPase. The use of siRNA or pharmacological tools to inhibit Na_V_1.2 expression or activity, respectively, prevented regeneration. On the contrary, the use of the sodium ionophore monensin, or the heterologous expression of human Na_V_1.5 in Na_V_1.2 knocked-out embryos were capable to restore tail regeneration. The sodium influx was therefore demonstrated to be critical and further identified as being responsible for activating downstream signaling such as the BMP and Notch pathways (Tseng et al., 2010).

Whether the modulation of the intracellular Ca^2+^ concentration is involved or not in this effect is not known. However, this study clearly highlights the role of intracellular Na^+^ as an important regulator of multiple signaling pathways at least in functions that appear relevant to the physiopathology of cancer.

### Current hypotheses on cellular mechanisms by which Na_v_ channels expressed in cancer cells promote invasiveness

The acquisition of an increased invasive capacity by cancer cells is a critical step in primary tumor growth and obviously in the development of metastases, especially in the case of carcinomas. What is known is that cancer cell invasiveness initially depends on the epithelial-to-mesenchymal transition (EMT), a process by which epithelial cancer cells lose their apico-basal polarity, cell-cell adhesion capacity, and gain migratory and invasive properties. Aggressive cancer cells are more invasive, owing to their ability to digest and to migrate through the extracellular matrix (ECM) (Gupta and Massague, 2006; Friedl and Alexander, 2011). Cancer cell motility relies on molecular mechanisms, such as the nucleation and polymerization of actin at the cell front, actomyosin contractility, and cycles of formation and disruption of focal adhesions. These mechanisms lead to the formation of lamellopodia at the cell front and the adhesion-mediated traction of the cell. All these properties are orchestrated by Rho guanosine triphosphate hydrolases and their associated signaling pathways (Friedl and Brocker, 2000; Ridley et al., 2003). The ECM proteolysis is performed through the activation of proteases, such as the matrix metalloproteinases (MMPs) or membrane-bound MMPs (MT-MMPs) (Egeblad and Werb, 2002) or the cysteine cathepsins (Jedeszko and Sloane, 2004; Mohamed and Sloane, 2006; Gillet et al., 2009), at the extracellular side of specialized structures of invasive cancer cells, protrusive into the ECM, and called invadopodia (Linder, 2007). The tumor microenvironment is also a determinant in cancer cells dissemination. Particularly, the extracellular compartment of tumors is known to be more acidic than the extracellular space of normal tissues (pH 6.2-6.8 instead of pH 7.2-7.4), owing to the selection, under hypoxic tumor conditions, of cancer cells having a preponderant glycolytic metabolism. Surprisingly, these cancer cells keep this preferred metabolism even in normoxic conditions, a condition called the “Warburg effect” which is associated with the intense efflux of metabolically-produced H^+^ in order to maintain the intracellular pH (pHi) (Cardone et al., 2005; Kroemer and Pouyssegur, 2008). The efflux of H^+^ generates the extracellular acidification that potentiates cell migration (Stock and Schwab, 2009) and enhances the degradation of the ECM for cancer cell dissemination (Cardone et al., 2005). Among all pH regulators, the Na^+^/H^+^ exchanger type 1 (NHE1) has drawn a lot of attention because it is overexpressed and over-activated in cancer cells (Gatenby et al., 2007), and its activity is associated with cancer cell survival, migration and metastatic progression (Cardone et al., 2005; Brisson et al., 2012). NHE1 was recently found to be localized in breast cancer cell invadopodia and to modulate both their formation and proteolytic activity (Frantz et al., 2007; Busco et al., 2010).

As detailed previously, Na_V_α are abnormally expressed in cancer cells, especially from epithelial origins, while they are not expressed in non-cancer tissues, and it is proposed that their function is associated with cancer progression (Roger et al., 2006; Brackenbury, 2012). Most of the mechanistic details have been obtained in breast cancer cells so far. Importantly, the expression of the Na_V_1.5 isoform in breast tumors, under the form of a neonatal splice variant called nNa_V_1.5, is associated with metastases development and patients' death (Fraser et al., 2005; Yang et al., 2012a). In highly aggressive human breast cancer cells, the activity of Na_V_1.5 is not associated with the triggering of action potentials. It enhances ECM degradation and cancer cell invasiveness by increasing the activity of extracellular acidic cysteine cathepsins B and S (Roger et al., 2003; Gillet et al., 2009). Cancer cell invasion is reduced by shRNA targeting the expression of Na_V_1.5, as well as by inhibitory molecules that plug and close the internal pore of the Na_V_ channel, such as tetrodotoxin, or reduce its activity such as ranolazine (Driffort et al., 2014). On the contrary, ECM invasion is increased by drugs that increase Na_V_ activity, such as veratridin (Gillet et al., 2009). Altogether, these results demonstrated the importance of Na_V_α channel activity, through a persistent window current and suggested the participation of a Na^+^-dependent signaling. Na_V_-dependent invasiveness has been shown to be mediated through the participation of NHE1 and the subsequent acidification of the pericellular microenvironment (Gillet et al., 2009; Brisson et al., 2011). NHE1 and Na_V_1.5 proteins can be co-immunoprecipitated and were proposed to form functional complexes located in caveolin-1-containing lipid rafts, and more specifically in invadopodia (Brisson et al., 2011, 2013). How Na_V_1.5 activity modulates NHE1 is unclear, and was initially counterintuitive as both transporters are expected to induce an entry of Na^+^ that would tend to slightly decrease the sodium gradient used as a fuel by NHE1 to extrude protons (See Figure 1). Therefore, even though it is very unlikely that the sodium gradient would be reversed, even in some subcellular microdomains, it could have been initially thought that Na_V_ activity would reduce the activity of NHE1. Instead, Na_V_1.5 was responsible for the allosteric modulation of the NHE1 exchanger, rendering it more active for the extrusion of H^+^ at pHi values between 6.4 and 7. Moreover, Na_V_1.5 activity sustained Src kinase activity, the phosphorylation of the actin-nucleation promoting factor cortactin on tyrosine 421, the polymerization of the F-actin cytoskeleton and the adoption by cells of a spindle-shaped elongated morphology (Brisson et al., 2013). Altogether, these results indicated a prominent role of Na_V_1.5 in both invadopodial formation and proteolytic activity, and its participation to “mesenchymal invasion” (Figure 1).

One working hypothesis is that the Na^+^ cation, by itself, is responsible for the allosteric modulation of NHE1 and the activation of members of the Src kinase family through a not yet identified Na^+^-dependent signaling pathway. Alternatively, Na_V_ activity could also induce local changes in intracellular Ca^2+^ concentration, possibly through NCX exchangers, which in turn could modulate the activity of NHE1, known to be modulated by intracellular Ca^2+^ through the binding of calmodulin to an IQ motif in its C-terminus (Li et al., 2013). These aspects will have to be further studied.

In two recent studies, performed by the group of Brackenbury and ours, it was clearly demonstrated that the expression of Na_V_1.5 in human breast cancer cells is critical for the metastatic colonization of lungs of immunodepressed mice, and that inhibitors of Na_V_1.5 that are approved by the Food and Drug Administration (FDA), such as ranolazine for the treatment of chronic angina, or phenytoin as an anticonvulsant, significantly reduced metastatic organ colonization by Na_V_1.5-expressing human breast cancer cells (Driffort et al., 2014; Nelson et al., 2015). These results certainly open new therapeutic opportunities in which conventional inhibitors of Na_V_ channels, already approved for other clinical use such as antiarrhythmic, anticonvulsant, or anesthetic drugs could be repurposed for the prevention and/or reduction of metastatic spreading from primary tumors.

## Conclusions

There is growing evidence that the occurrence of metastatic carcinomas is associated with the abnormal expression of Na_V_. Several different Na_V_α have been particularly identified such as Na_V_1.5, Na_V_1.6, and Na_V_1.7. Not only are these proteins expressed but they are also active, giving rise to sodium currents which appear to be influential for the regulation of invasive properties, in a manner that is most likely independent of the generation of action potentials. While the protein is important, its Na^+^ permeation might be the key parameter controlling signaling pathways of the mesenchymal invasion, through the regulation of different other proteins such as NHEs or NCXs. Interestingly, there is also new evidence indicating that Na_V_α are involved in the development and regeneration of organs during embryogenesis through pathways that are independent of membrane excitability. These results suggest that Na_V_ proteins may have many physiological roles. They might represent important regulators of the sodium homeostasis being involved in more cellular properties than the sole control of the membrane potential, and that will have to be further studied.

**Figure 2:**
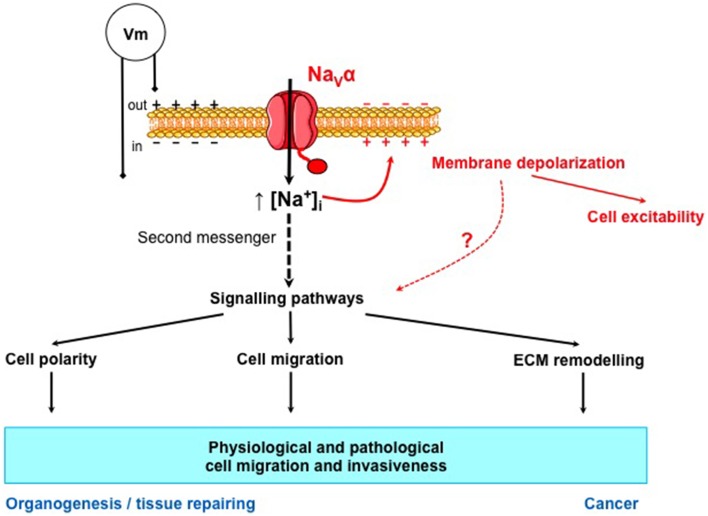
**Na_V_α proteins control the intracellular concentration of Na^+^, a second messenger that could control normal and cancer cell migration and invasiveness**. Na_V_α proteins allow the entry of Na^+^ into the cell. This entry of cations is known to depolarize the membrane potential (Vm) and to initiate action potentials in excitable cells. Na_V_α proteins also controls the intracellular concentration of Na^+^ that could be considered as a second messenger since it regulates, directly or indirectly, many signaling pathways involved in cell polarity, cell migration, and extracellular matrix (ECM) remodeling. These properties are involved in physiological cell migration and invasiveness, such as in organogenesis and tissue repairing and also in the pathological migration and invasiveness of cancer cells.

If we postulate that in cancer, the abnormal expression of proteins is the consequence of the re-expression of a fetal or neonatal phenotype, it is likely that Na_V_ were involved in the initial migration and invasiveness of cells during the development of organs. If so, this suggests that during development of organisms, Na_V_ may have different roles: a primary role in embryogenesis and organogenesis independent of cell excitability of not yet differentiated cells, and secondarily in the generation of action potentials and rapid cell-cell electrochemical communications in well-differentiated cells such as nerve or muscle cells. Eventually, we propose that Na^+^ ions are important second messengers that are finely regulated in time and intracellular concentration in normal cells, and deregulated in the cancer disease (Figure 2).

### Conflict of interest statement

The authors declare that the research was conducted in the absence of any commercial or financial relationships that could be construed as a potential conflict of interest.

## Abbreviations

CAM: cell adhesion molecules
CNS: central nervous system
ECM: extracellular matrix
Ig: immunoglobulin
INa: sodium currents
LQT: long Q-T syndrome
MMP: matrix metalloproteinases
NaV: Voltage-gated sodium channels
NaVα: pore-forming α subunit of voltage-gated sodium channels
NaVβ: β subunits of voltage-gated sodium channels
NCX: sodium-calcium exchanger
NHE: sodium-proton exchanger
PNS: peripheral nervous system
TTX: tetrodotoxin
VSD: voltage-sensing domain.
